# Effect of Elemental Iron Containing Bauxite Residue Obtained After Electroreduction on High-Pressure Alkaline Leaching of Boehmitic Bauxite and Subsequent Thickening Rate

**DOI:** 10.3390/ma18020224

**Published:** 2025-01-07

**Authors:** Andrei Shoppert, Irina Loginova, Malal Mamodou Diallo, Dmitrii Valeev

**Affiliations:** 1 Laboratory of Advanced Technologies in Non-Ferrous and Ferrous Metals Raw Materials Processing, Ural Federal University, 620002 Yekaterinburg, Russia; 2 Department of Non-Ferrous Metals Metallurgy, Ural Federal University, 620002 Yekaterinburg, Russia; i.v.loginova@urfu.ru (I.L.); malalkoura2@gmail.com (M.M.D.); 3 Laboratory of Sorption Methods, Vernadsky Institute of Geochemistry and Analytical Chemistry of the Russian Academy of Sciences, 119991 Moscow, Russia; 4 College of Environmental Science and Engineering, UNEP-TONGJI Institute of Environment for Sustainable Development, Tongji University, Shanghai 200092, China

**Keywords:** red mud, Bayer process, reductive leaching, electrolytical reduction, magnetite, waste utilization

## Abstract

The use of reduction leaching in the production of alumina from bauxite by the Bayer process in order to decrease the amount of waste (bauxite residue) by adding elemental iron or aluminum, as well as Fe^2+^ salts and organic compounds in the stage of high-pressure leaching, requires the purchase of relatively expensive reagents in large quantities. The aim of this study was to investigate the possibility of the use of electrolytically reduced bauxite residue (BR) as a substitute for these reagents. Reduced BR was obtained from Al-goethite containing BR using a bulk cathode in alkaline suspension. The degree of deoxidation of Fe^3+^ compounds was 55% after 2 h of electrolysis with a current yield of more than 73%. The addition of reduced BR according to the shrinking core model leads to a change in the limiting stage of the high-pressure boehmitic bauxite leaching from a surface chemical reaction to internal diffusion. The activation energy decreased from 32.9 to 17.2 kJ/mol by adding reduced red mud. It was also shown that the addition of reduced BR increased the rate of thickening of the slurry after leaching by a factor of 1.5 and decreased the Na_2_O losses by 15% without the addition of lime. The solid residue was examined by means of X-ray diffraction analysis and scanning electron microscopy to confirm the presence of magnetite and elemental iron. A preliminary techno-economic analysis was carried out to assess the applicability of the proposed process.

## 1. Introduction

In the treatment of bauxite to produce alumina by the Bayer process, the feedstock is subjected to high-pressure leaching with a recycled alkaline–aluminate solution [1,2,3] to produce a saturated solution and a solid leach residue—bauxite residue (BR) [4,5,6]. The BR consists mainly of unreacted iron and titanium minerals as well as solution desilication products (DSP, Na_6_[Al_6_Si_6_O_24_] Na_2_O) [7,8,9] formed by Equations (1) and (2).

As a result, the BR consists of approximately 50% iron oxide, with the remainder being DSP and other impurities [10,11,12]. The yield of BR can be up to 100% of the weight of the raw bauxite, especially when the addition of CaO is high [13,14,15]. The addition of CaO is necessary to reduce losses of alkali due to the formation of hydrogarnet (Ca_3_Al_2_Si_3_(OH)_12_) by reaction (2) [16,17,18]. It also increases the sedimentation properties of the BR, making it easier to separate solid particles from the aluminate solution [19,20]. As a result of the formation of DSP and the artificial introduction of lime, the iron content of the red mud is decreased (up to 35%), caustic consumption is increased (up to 100 kg/t of alumina), and alumina recovery is lower than 80% [21]. Consequently, the red mud generated by the existing Bayer technology is not suitable for the extraction of iron and valuable elements, including rare earth elements, the content of which in red mud is several times greater than the average value for the earth’s crust [22].

Li et al. [23,24,25] have shown that, in the leaching of bauxite with the addition of iron, aluminum, or organic powders to establish reduction conditions, there is no need to use calcium. In addition, the extraction of Al in solution is increased, and the yield of BR is reduced due to the absence of CaO addition. The process is based on the conversion of hematite in bauxite to magnetite by reaction (3). This reaction occurs in the presence of Fe^2+^ ions in solution [26,27].

6NaAl(OH)_4_ + 6Na_2_SiO_3_ = Na_6_[Al_6_Si_6_O_24_] Na_2_O + 10NaOH + 7H_2_O, (1)

2NaAl(OH)_4_ + 3Na_2_SiO_3_ + 3CaO + 10H_2_O = Ca_3_Al_2_Si_3_(OH)_12_ + 8NaOH + 8OH^-^, (2)

Fe_2_O_3_ + Fe(OH)_3_^−^ = Fe_3_O_4_ + 4H_2_O. (3)

Previous studies [21,28] have shown that reductive leaching can be performed at atmospheric pressure by adding FeSO_4_. This produces a red mud that is rich in iron with magnetite as the main mineral. The solid residue was found to be easier to separate from the aluminate solution and to thicken faster. This is due to the finalization of the processes for the extraction of aluminum from the aluminogoethite (Al-goethite) and the aluminohematite (Al-hematite). At the same time, it is shown in [29] that Al-goethite and Al-hematite matrices can be easily destroyed using electrochemical recovery. In this case, in addition to magnetite, the BR also contains elementary iron that results in reductive conditions during high-pressure leaching.

In this research, an attempt was made to use electrochemically reduced BR for high-pressure Bayer leaching (HPBL) of boehmitic bauxite without adding CaO for alumina extraction simultaneously with Al-goethite and Al-hematite dissolution, as well as to increase the rate of thickening of the BR particles.

The effect of technological parameters, such as time and temperature on the extraction of Al from boehmitic bauxite with the addition of reduced BR or CaO was studied. The experimental data that was obtained was processed using a shrinking core model to examine the leaching kinetics and establish the change in the mechanism of the HPBL process. The pulp from HPBL leaching was subjected to thickening tests to study the effect of reduced BR addition on the sedimentation rate. The raw bauxite and BR were studied using scanning electron microscopy with energy dispersive analysis (SEM-EDS), X-ray phase analysis (XRF), and X-ray diffraction analysis (XRD). The addition of RBR at the HPBL results in the decrease of Na_2_O losses with BR. Furthermore, it is shown that the sedimentation properties of BR were significantly enhanced after leaching with the RBR.

## 2. Materials and Methods

### 2.1. Materials and Reagents

**Figure 1:**
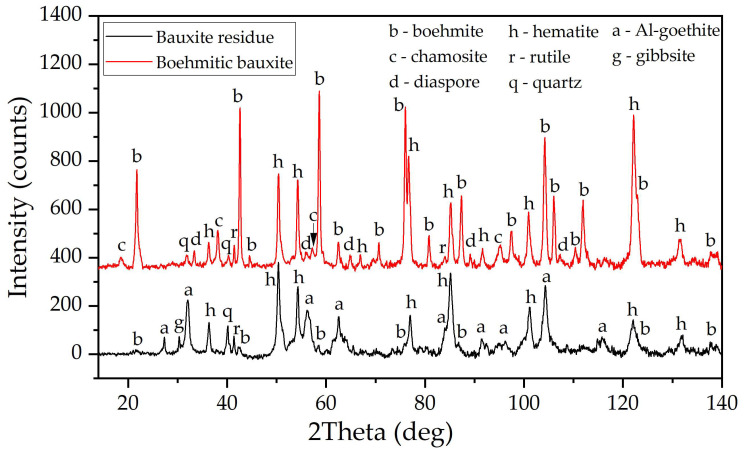
XRD patterns of the raw bauxite and bauxite residue (BR).

Boehmitic bauxite was collected from the Ural alumina refinery (Sverdlovsk region, Russia). Al-goethite containing bauxite residue was obtained from the Friguia alumina refinery (Guinea), which utilizes gibbsite bauxite for alumina production by the Bayer method. The chemical composition of the bauxite and BR is given in Table 1. The XRD of boehmitic bauxite and BR is shown in Figure 1.

The raw bauxite consisted of boehmite (AlOOH), hematite (Fe_2_O_3_), rutile (TiO_2_), quartz (SiO_2_), and chamosite ((Fe^2+^,Mg,Al,Fe^3+^)_6_(Si,Al)_4_O_10_(OH,O)_8_). The BR consisted of Al-goethite, hematite, gibbsite, and desilication product. The presence of Al-goethite and Al-hematite in this BR was confirmed by Mössbauer spectrometry [21]. Analytical-grade NaOH for solution preparation was purchased from JSC Soda (Sterlitamak, Russia). The mother liquor for HPBL was obtained from the Ural Alumina Refinery and contained 280.6 g L^−1^ caustic alkali (Na_2_O), 35.5 g L^−1^ sodium carbonate (Na_2_CO_3_) and 130.4 g L^−1^ Al_2_O_3_. Prior to leaching, the bauxite was ground until 80% of the particles were less than 74 µm in size. To evaluate the leaching kinetics, the bauxite was sieved to obtain a narrow size fraction between 50 µm and 74 µm.

### 2.2. Analytical Methods

XRD analysis of raw materials and solid products was performed using a Difrei-401 X-ray diffractometer (JSC Scientific Instruments, Saint-Peterburg, Russia) with a Cr-Ka radiation source and 30 min exposure time. The phase composition of the samples was determined using Match! 3 software (Crystal Impact, Bohn, Germany). Solid products, particle morphology, and elemental mapping were defined by SEM-EDX scanning electron microscopy Vega III (Tescan, Brno, Czech Republic). The chemical composition of the samples was determined using an EDX-8000 powder X-ray fluorescence spectrometer (Shimadzu, Kyoto, Japan).

### 2.3. Bauxite Residue Pretreatment Using Electrolysis

Al-goethite containing BR was subjected to electrolytic reduction in a 500 mL stainless-steel thermostated reactor coated with PTFE (polytetrafluoroethylene) by the method described in previous research [29] using a bulk cathode. A total of 90 g of BR and 300 mL of hot caustic alkaline solution at a concentration of 330 g L^−1^ were added to the reactor prior to reduction. After vigorous stirring, the pulp was thickened and heated to 110 °C within 30 min before electrolysis. The bulk cathode consisted of a round stainless-steel mesh with a diameter of 9.2 cm (surface area 110 cm^2^), and the BR particles thickened on its surface. The mesh was connected by stainless-steel wire to the terminal of a ZiveLab SP 2 potentiostat (Zivelab, Seoul, Korea). A 10 cm^2^ nickel plate was used as the anode. The reference electrode was a mercury/mercury oxide electrode OH-/HgO, Hg (RE-1A, YM, Shaanxi, China). The electrolysis process was carried out at a constant current of 1.98 A and temperature of 110 °C for 2 h (with a theoretical reduction degree of Fe^3+^ compounds to magnetite according to Equation (3) and the electrochemical equivalent [29] = 74.8%). For the potentiodynamic measurement, the amount of BR was reduced to 30 g.

### 2.4. Experimental

Leaching experiments were performed in hermetically sealed steel reactors placed in an air thermostat (JSC EKROS, Saint-Petersburg, Russia) with mixing through the head. The stirring speed in all experiments was 40 rpm. A mixture of bauxite and additives (reduced BR and CaO) with Bayer mother solution with a solid–liquid ratio (S/L) to obtain a molar ratio of Na_2_O to Al_2_O_3_ in the pregnant solution of 1.65. In the high-pressure kinetic leaching experiments, the temperature was 190, 210, 230, and 250 °C; the leaching time was 15, 30, 45, 60, and 90 min with the addition of reduced BR in the amount of 10% of the bauxite mass and CaO in the amount of 3% of the bauxite mass and without addition. Prior to the thickening tests, the bauxite was leached at 230 °C for 90 min. The pulp obtained after leaching was diluted twice and thickened in a cylindrical glass beaker placed in an air thermostat at 95 °C with linear graduation in mm in order to evaluate the thickening efficiency. The underflow obtained after thickening was filtered, and the sample was washed with hot water and dried at 105 °C for 4 h. Then, the obtained BR was weighed and analyzed, and the Al extraction degree was calculated using Equation (4).

X = (m_1_ × X_1_ − m_2_ × X_2_)/(m_1_ × X_1_), (4)

where m_1_ is the original sample amount (g); X_1_ is the element content in the raw bauxite (%); m_2_ is the leaching residue amount (g); X_2_ is the element content in the solid residue (%).

## 3. Results and Discussion

### 3.1. Electroreduction of Al-Goethite Containing Bauxite Residue

**Figure 2:**
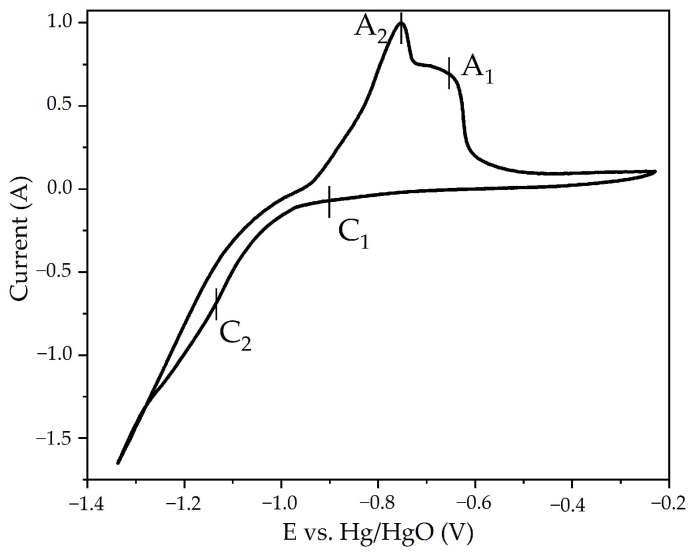
Cyclic voltammetry (CV) at a temperature of 110 °C and a solid concentration of 100 g L^−1^ for electrolytic reduction in a suspension of Al-goethitic BR in alkaline solution with a concentration of 330 g L^−1^ Na_2_O.

In order to evaluate the potential at the cathode during the electrolytic reduction of iron oxide in the suspension of Al-goethite containing BR in an alkaline solution, which is necessary to obtain magnetite and elemental iron in the product, voltametric measurements were carried out at 110 °C and an S/L ratio of 1:10 (Figure 2).

According to Figure 2, there are two distinct anodic peaks and only one cathodic peak. The current C_1_ with a potential of ~−0.9 V, which was caused by the formation of Fe^2+^ compounds [30], was not detected in these measurements. It may be related to the use of goethitic BR instead of hematitic in previous research [29] and the bulk cathode. The current C_2_ at E = −1.16 V can be attributed to the reduction of iron compounds to Fe [31]. At cathodic potentials greater than 1.2 V, the side reaction of hydrogen evolution became dominant. Peak A_1_, visible as a shoulder at a potential of −0.7 V, is attributed to the oxidation of Fe^2+^ compounds formed in the region of peak C_1_ [31]. Peak A_2_ refers to the oxidation of Fe to Fe^2+^.

**Figure 3:**
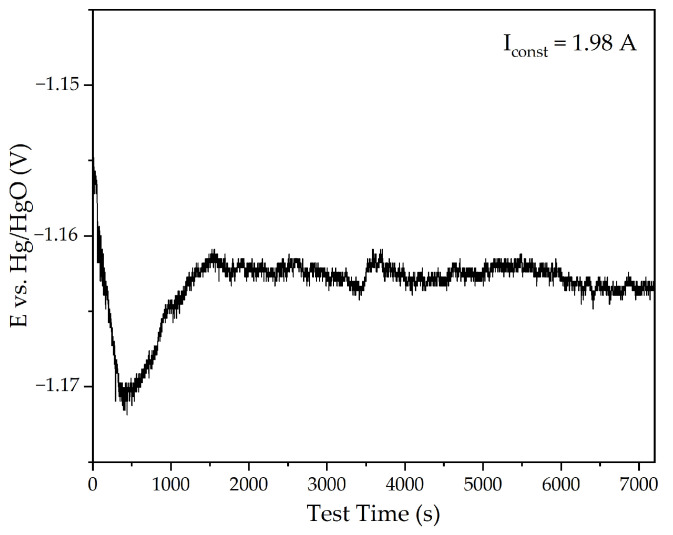
The dependence of the potential at the cathode on electrolysis time at a constant current of 1.98 A using the bulk cathode and the suspension of BR in alkaline solution.

CV measurements confirmed the formation of Fe^2+^ compounds and Fe using the bulk cathode and the Al-goethite containing BR. The potential at the cathode should be maintained at −1.16 V. Using the 110 cm^2^ power supply, this potential was achieved at I_const_ = 1.98 A and a solid concentration of 300 g L^−1^ (Figure 3).

**Figure 4:**
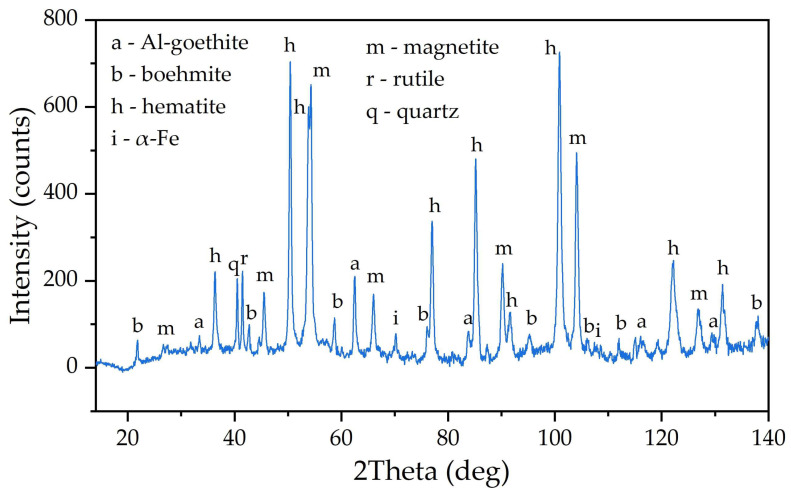
XRD pattern of the reduced BR.

Initially, an increase in the cathodic potential was observed, which could be explained by the deterioration of the contact between the BR particles and the current supply because of hydrogen evolution. After 1500 s of electrolysis, the potential at the cathode decreased to −1.162 V. The decrease in overvoltage can be attributed to the increase in the cathode area caused by the formation of iron on the surface of the power supply and the further thickening of the BR particles. The XRD diagram of the reduced BR is shown in Figure 4.

**Figure 5:**
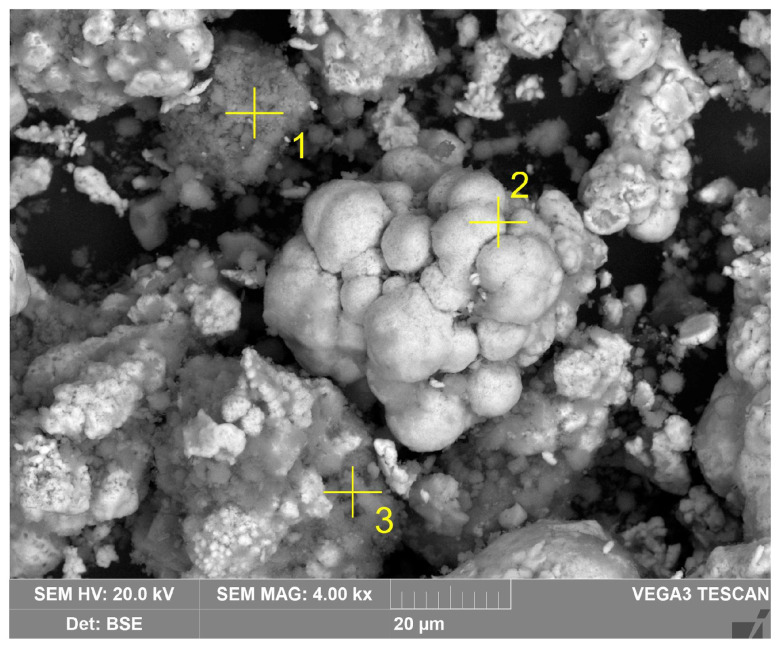
SEM image of reduced BR with the point of SEM-EDX analysis (the corresponding element composition is listed in Table 2).

As can be seen in Figure 4, almost all of the Al-goethite was leached from the BR during electrolysis. After reduction, two new phases appeared: magnetite and α-Fe. SEM-EDX analysis (Figure 5 and Table 2, spectrum 2) confirmed the presence of elemental iron on the surface of the particles. Boehmite and desilication products can also be seen (spectra 1 and 3 in Table 2).

The chemical composition of the reduced BR is shown in Table 3. The amount of Al was greatly reduced, confirming the dissolution of Al-goethite and desilication product. After the dissolution of Al and Si and the reduction of LOI, the Fe_2_O_3_ content was greatly increased from 52.5% to 77.4%.

The BR obtained after filtration and washing was subjected to Bayer process leaching at 250 °C for 90 min to convert iron and residual hematite to magnetite. The degree of deoxidation of Fe^3+^ minerals to magnetite after electrolysis and Bayer leaching was 55%. Therefore, the proportion of current used for magnetite formation was more than 73.8%, since at 100% current efficiency there should be a 74.8% reduction as shown in Section 2.3.

### 3.2. Effect of Addition of Reduced Bauxite Residue on Al Extraction

**Figure 6:**
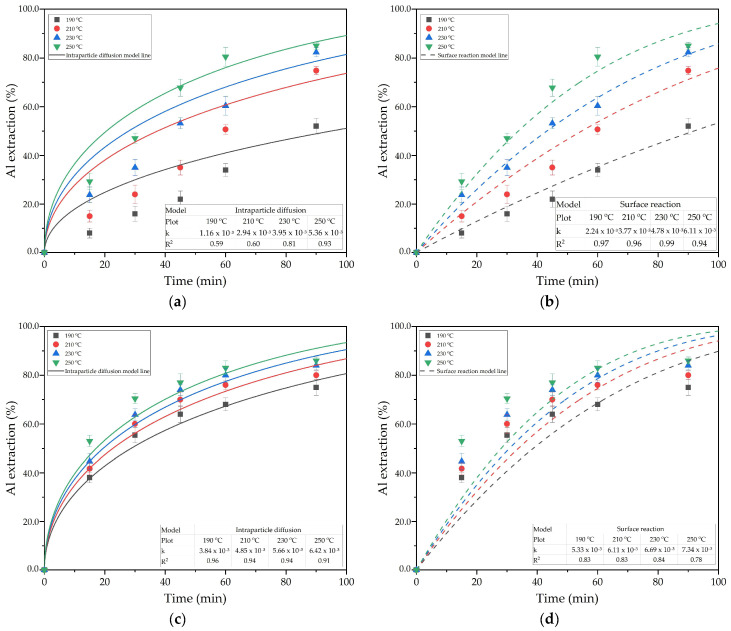
The effect of leaching time and temperature (symbols) on the following: (**a**) Al extraction from bauxite with the addition of CaO (intraparticle diffusion model); (**b**) Al extraction from bauxite with the addition of CaO (surface reaction model); (**c**) Al extraction from bauxite with the addition of reduced BR (intraparticle diffusion model); (**d**) Al extraction from bauxite with the addition of reduced BR (surface reaction model). The non-linear fitting of data into Equation (5)—solid lines—and Equation (6)—dash line.

The Bayer process leaching tests at different temperatures and leaching times were used to assess the effect of the addition of reduced BR and lime on Al extraction from boehmitic bauxite (Figure 6). The addition of lime in the Bayer process helps to reduce the alkali loss with the BR and increases the thickening rate of the resulting pulp. However, as shown in Figure 6, there is a marked change in the mechanism of bauxite leaching with the addition of reduced BR and lime. In order to identify the limiting stage of the process with different additives, non-linear fitting of experimental data into shrinking core models [32,33] was used. The use of this method helps to distinguish the limiting stage by means of curve characteristics. When the limiting step is the diffusion of the solution through the porous product of the reaction, there is a marked decrease in the reaction rate due to the increase in the product layer, and the process can be described by Equation (5):

[1 − 2/3X − (1 − X)^2/3^] = kt. (5)

where X is the share of leaching; k is the rate constant, 1/s; t—time, s.

If the limiting stage is the surface chemical reaction, the reaction rate is reduced by the change in the radius of the unreacted core. This decrease in the leaching rate is less pronounced, and the process can be described by Equation (6):

[1 − (1 − X)^1/3^] = kt, (6)

When the process is limited by external diffusion, there is no correlation between the radius of the unreacted core and the reaction rate. Therefore, the process can be described by Equation (7).

X = kt,(7)

According to the results of the non-linear fitting in Figure 6, the limiting stage of the process has been changed with the addition of reduced BR. The surface chemical reaction itself is much faster, which may be related to the destruction of Al-goethite and Al-hematite in the presence of Fe^2+^ species in solution [34]. The presence of Al-goethite and Al-hematite in this type of bauxite was confirmed by Mössbauer spectrometry in our previous research [35].

**Figure 7:**
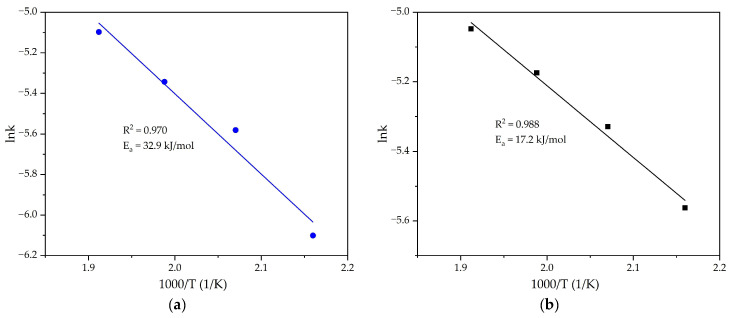
Dependence of (**a**) logarithm k in Figure 6b and (**b**) logarithm k in Figure 6c on inverse temperature.

To evaluate the activation energy of the process, the linear form of the Arrhenius Equation (8) and the values of the rate constant obtained at different temperatures in Figure 6 were used. The results of the substitution are shown in Figure 7. With the addition of the reduced BR, the activation energy was reduced from 32.9 to 17.2 kJ/mol, confirming the change in the limiting stage.

lnk = lnA + Ea/RT,(8)

The presence of Al-goethite and Al-hematite in bauxite is known to result in increased persistence for the extraction of Al, as high temperatures and the addition of lime are required to dissolve them. This explains the higher activation energy values for Al extraction from bauxite when lime alone is added (Figure 7a). The use of reductive leaching removes the kinetic constraints due to phase transformations of iron compounds [21], resulting in lower activation energies (Figure 7b), as the process starts to be limited by reagent access within the particles.

### 3.3. Effect of Addition of Reduced Bauxite Residue on Thickening of the Pulp

**Figure 8:**
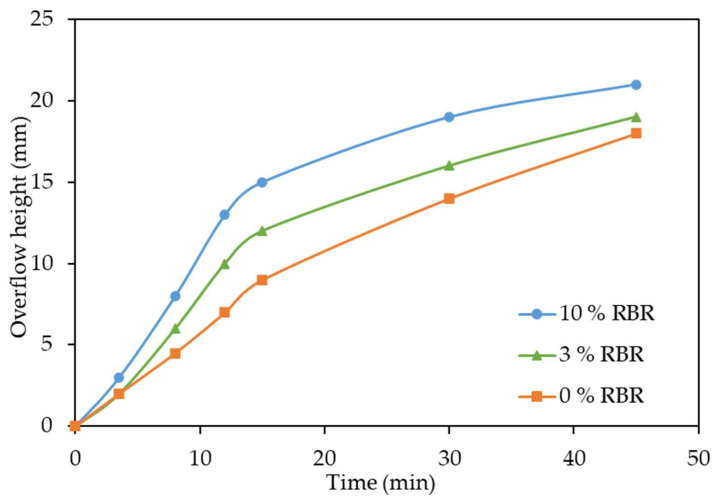
Effect of addition of reduced BR on thickening rate without lime.

The thickening tests were carried out to compare the sedimentation of the BR obtained after high-pressure leaching of boehmitic bauxite with the addition of reduced BR and without any addition under the same conditions: T = 230 °C, a leaching time of 1.5 h, an S/L ratio to obtain a Na_2_O/Al_2_O_3_ molar ratio in the pregnant solution of 1.65. Before thickening, the pulp was diluted twice with hot water in order to increase the thickening rate by reducing the viscosity. The results of these tests are shown in Figure 8.

It was found that the addition of magnetite and iron-containing BR in the leaching process of bauxite in the amount of 3–10% of the bauxite mass without lime, all other conditions being equal, significantly increases the thickening rate (Figure 8).

It can be seen that the addition of reduced BR in the amount of 3% of the bauxite mass increases the thickening rate by a factor of 1.33, as the height of the clear solution layer after 15 min of thickening (first linear stage) increases from 9 to 12. After the addition of the reduced BR in the amount of 10% of the bauxite mass, the thickening rate increased by a factor of 1.66 (the height of the linear stage increases from 9 to 15 mm). The effect of reduced BR is associated with the formation of a new ferrous phase in the solid residue, dissolution of Al-goethite and Al-hematite, and formation of the low-alkali desilication product [36].

**Figure 9:**
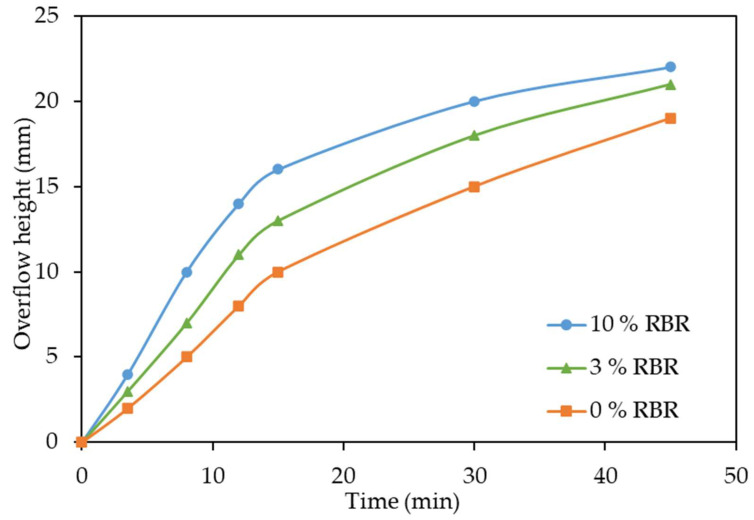
Effect of addition of reduced BR on thickening rate in the presence of 3% CaO.

After high-pressure leaching of bauxite with the addition of lime together with magnetite and iron-containing BR in the amount of 10% of the bauxite mass, the thickening rate increases up to 1.7 times (Figure 9). However, the thickening rate without the addition of reduced BR or with the addition of 3% reduced BR was also greatly increased by the addition of lime, which also enhances the sedimentation due to the formation of new DSP and a higher degree of Al-goethite and Al-hematite dissolution.

### 3.4. Effect of Addition of Reduced Bauxite Residue on the Chemical Composition of Solid Residue and Na_2_O Losses

**Figure 10:**
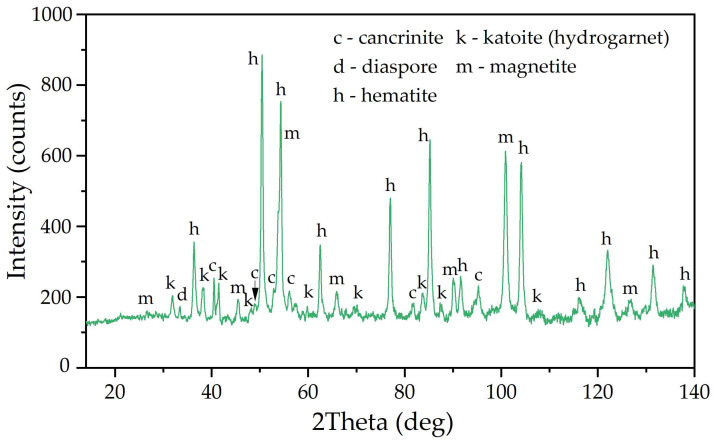
XRD pattern of the solid residue after bauxite leaching with the addition of the reduced BR and lime.

It was also found that the addition of magnetite and iron-containing BR in the boehmitic bauxite leaching process in the amount of 10% of the bauxite mass without the use of lime, all other conditions being equal, allows the Na_2_O losses with the solid residue to be decreased by 15% (Table 4). With the addition of lime and reduced BR, Na_2_O losses were decreased by 17%, all other things being equal, even though the Na_2_O content was decreased from 5.3% to 4.6% (i.e., by 13.2%). This is due to the higher yield of the BR with the addition of lime due to the formation of katoite (Ca_3_Al_2_Si_3_(OH)_12_) (Figure 10).

The higher yield of BR with the lime addition also results in lower Fe_2_O_3_ content in BR and lower Al extraction (87.4% vs. 88.5%).

The Fe_2_O_3_ content in the solid residue of bauxite leaching with BR containing magnetite and iron is 51–52% compared to 46% Fe_2_O_3_ after conventional leaching according to the Bayer process. According to the XRD diagram in Figure 10, the main phases of iron in the solid residue of leaching with RBR are hematite and magnetite in proportions of about 60% and 40%, respectively. The presence of magnetite allows the extraction of a magnetic fraction from this residue by magnetic separation [37,38,39].

### 3.5. Evaluation of the Feasibility of the Process

**Figure 11:**
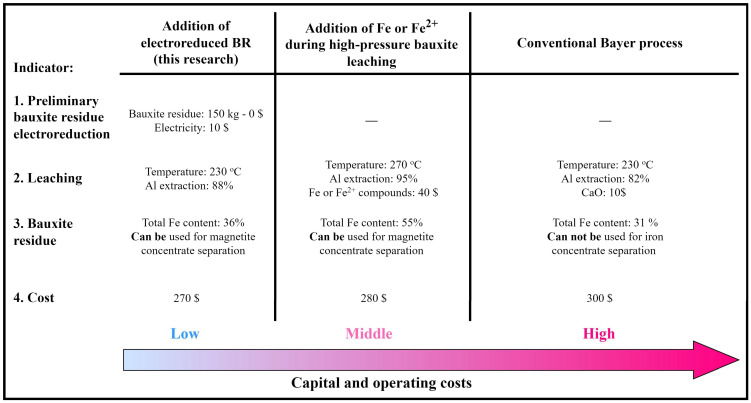
Techno-economic analysis of bauxite leaching with the addition of the reduced BR (data for the addition of Fe or Fe^2+^ during high-pressure bauxite leaching are taken from [40]).

To assess the feasibility of the process of adding electrolytically reduced BR, a techno-economic analysis (Figure 11) was performed by comparing the cost of producing a ton of Al_2_O_3_ using different methods, taking into account the Al extraction rate.

The relative information is represented by a linear dependence on the increase in cost from the lowest to the highest. The reduced BR method is considered in this paper to be a lower-cost method because it uses electrochemically produced iron and magnetite directly from the BR, and the pressure leach process itself follows the same parameters as the standard Bayer process. Other methods, such as adding Fe or Fe^2+^ compounds, require the use of a third-party iron source, resulting in significantly higher reagent costs, although this method produces the most iron-rich BR with higher Al extraction [40]. However, in addition to the purchase of reagents, this method requires the use of more expensive and highly reliable equipment. This is because the process takes place at 270 °C with high hydrogen evolution. As a result, a significant capital investment will be required. The conventional Bayer process requires the addition of large quantities of lime, which significantly increases the yield of BR that is not suitable for iron concentrate separation and is also poorly thickened. This suggests that the addition of reduced BR provides an optimum balance between capital and production costs to produce a BR suitable for subsequent magnetite concentrate separation. Further research will be focused on the investigation of the mechanisms underlying the effect of the reduced BR addition on the pressure leaching and subsequent thickening process using Mössbauer spectrometry and zeta potential measurements, which will help to further improve the efficiency of the process.

## 4. Conclusions

The effect of electrolytically reduced BR addition on high-pressure alkaline leaching of boehmitic bauxite and following the thickening rate was investigated in this study. The main conclusions are as follows:

1. The electrolytic reduction of iron minerals in the suspension of Al-goethite containing BR in an alkaline solution using a bulk cathode results in more than a 55% reduction degree after 2 h with the current efficiency higher than 73%.
2. The addition of reduced BR at the level of 10% of the bauxite mass leads to a change in the leaching mechanism. The activation energy was decreased from 32.9 to 17.2 kJ/mol. The limiting stage according to the shrinking core model changed from a surface chemical reaction to intraparticle diffusion due to easier Al extraction from Al-goethite and Al-hematite in the presence of Fe^2+^ ions.
3. After high-pressure leaching with the addition of 3% reduced BR, the thickening rate increases by 1.33 times. Leaching of boehmitic bauxite with the addition of lime and reduced BR in the amount of 10% of the bauxite mass increases the thickening rate up to 1.7 times.
4. Leaching of boehmitic bauxite with the addition of lime and reduced BR in the amount of 10% of the bauxite mass leads to Na_2_O losses being reduced by 15%.
5. The Fe_2_O_3_ content in the solid residue of boehmitic bauxite leached with the addition of reduced BR reaches 50–52% against 46% in conventional leaching according to the Bayer process.
6. Techno-economic analysis suggests that the addition of reduced BR provides an optimum balance between capital and production costs to produce a BR suitable for subsequent magnetite concentrate separation.

## Figures and Tables

**Table 1:** Chemical composition of the raw boehmitic bauxite and bauxite residue (BR) from Friguia alumina refinery, wt. %.

Sample	Fe_2_O_3_	Al_2_O_3_	SiO_2_	TiO_2_	Na_2_O	CaO	MgO	SO_3_	P_2_O_5_	Other	LOI ^1^
Bauxite	25.5	51.7	6.4	2.7	0.1	0.5	0.4	0.02	0.3	1.1	11.3
BR	52.5	19.0	7.7	3.1	3.8	0.2	0.4	0.3	0.3	1.2	11.5

^1^ Lost on ignition at 1100 °C.

**Table 2:** Elemental compositions (wt.%) of reduced BR particles (the spectral numbers correspond to the areas highlighted in Figure 5).

Spectrum	1	2	3
O	55.2	2.6	44.5
Al	31.9	0.8	16.7
Si	0.1	0.3	14.4
Fe	8.4	96.1	13.0
Ti	1.0	0.3	0.6
Na	2.9	0.1	13.0

**Table 3:** Chemical composition of reduced BR, wt. %.

Sample	Fe_2_O_3_	Al_2_O_3_	SiO_2_	TiO_2_	Na_2_O	CaO	MgO	SO_3_	P_2_O_5_	Other	LOI ^1^
Reduced BR	77.4	4.4	4.9	4.5	3.0	0.2	0.3	0.4	0.1	0.4	4.5

^1^ Lost on ignition at 1100 °C.

**Table 4:** The chemical composition of BR obtained after boehmitic bauxite leaching with the addition of lime and reduced BR, wt. %.

Sample	Fe_2_O_3_	Al_2_O_3_	SiO_2_	TiO_2_	Na_2_O	CaO	MgO	SO_3_	MnO	Other	LOI ^1^	Na_2_O Losses, kg/t Al_2_O_3_
Without additive	45.9	13.8	14.3	5.7	6.8	2	1.5	1.6	1.1	1.3	6.0	48.4
10% RBR	51.8	12.5	13.1	5.8	5.3	1.6	1.2	1.5	1	1.0	5.1	41.1
10% RBR + 3% CaO	50.5	12.2	12.9	5.7	4.6	4.8	1.1	1.5	0.9	1.0	4.9	40.3

^1^ Lost on ignition at 1100 °C.

## Data Availability

The original contributions presented in the study are included in the article, and further inquiries can be directed to the corresponding author.
